# The duality of retail pharmacy clinics in Mexico: from threat to opportunity

**DOI:** 10.1016/j.lana.2025.101258

**Published:** 2025-09-26

**Authors:** Diego Ramonfaur, Moises Auron, Guillermo Torre-Amione

**Affiliations:** a Department of Internal Medicine, Cleveland Clinic, Cleveland, OH, USA; b Department of Hospital Medicine, Cleveland Clinic, Cleveland, OH, USA; c Tecnológico de Monterrey, Escuela de Medicina y Ciencias de La Salud, Monterrey, Mexico

Despite the promise of universal healthcare, Mexico’s public health system remains overwhelmed and under-resourced, leaving millions without timely and affordable care. In response, an informal parallel system has emerged in the past two decades: the *retail pharmacy clinic*. These ambulatory sites, typically co-located with private pharmacies, represent a distinct form of retail clinic built on a vertically integrated model that consolidates both diagnostic services and medication dispensing under a single commercial entity. Retail pharmacy clinics provide low-cost medical consultations and have become a cornerstone of low-acuity healthcare delivery across socioeconomic strata.^1^ This model is common in low- and middle-income countries, and helps fill gaps left by a constrained public health infrastructure, though potentially introducing commercial conflicts of interest in prescribing practices.

Although retail pharmacy clinics are not officially part of Mexico’s national health system, they have become indispensable, particularly since the dissolution of *Seguro Popular*^2^ and the added burden brought by the COVID-19 pandemic.^3^ Retail pharmacy clinics offer convenience, affordability, and accessibility. Visits typically cost $2–5 USD, with some services provided for free. These clinics attract hundreds of millions of visits annually, reflecting both high demand and a profound unmet need in the public sector. Despite their prominence, retail pharmacy clinics operate in a regulatory gray zone and remain largely ungoverned in terms of quality, outcomes, and economic sustainability. Data-driven oversight and research into clinical outcomes, prescribing behaviors, and patient satisfaction are critical to avoid reactive and uncoordinated policy responses.

Misalignment of financial incentives with clinical care is particularly concerning (Fig. 1). Physicians in retail pharmacy clinics experience deplorable working conditions,^4^ and often face pressure to prescribe medications to meet sales quotas,^5^ raising significant ethical concerns. Population studies suggest that patients visiting these clinics are prescribed more active substances per visit than those in public or private institutions, implying systematic overprescription.^6^ In this model, diagnosis and dispensing are vertically integrated, enhancing profitability but potentially undermining rational prescribing and patient safety as overprescribing may contribute to polypharmacy, out-of-pocket expenditure, adverse drug reactions, and poor antibiotic stewardship.^6^ Regulation of antibiotic dispensing in physician offices has proven effective in reducing overprescription in other countries.^7^ The financial success of retail pharmacy clinics in Mexico is rooted in a weak public health infrastructure and a high out-of-pocket healthcare burden, currently among the highest in Latin America.^8^ In the United States, major retail chains like Walmart, and Walgreens have withdrawn from similar models, hindered by challenges in insurance reimbursement and the complexity of integrating with traditional healthcare systems. Interestingly, CVS’ MinuteClinic provides more promise as a vertically integrated model with insurance is employed.^9^

**Fig. 1:**
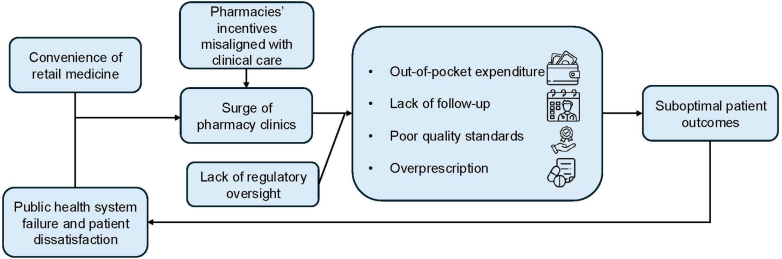
Systemic drivers and consequences of pharmacy clinics in Mexico.

The swift elimination of retail pharmacy clinics in Mexico is not currently feasible and would likely trigger a collapse of healthcare access for large swaths of the population. Yet their continued operation without regulatory oversight risks entrenching low-quality care, overuse of medications, and fragmented services. Retail pharmacy clinics face the duality of being a safety net and a risk, which warrants immediate policy attention.

Retail pharmacy clinics are primarily oriented toward episodic and urgent low-acuity care.^10^ This misses the opportunity to leverage their extensive geographical coverage and an established patient base to offer preventive, longitudinal, and community-based primary care if adequately supported and regulated. To realize this potential, a public-private partnership model must be explored. Mexico’s Secretariat of Health could integrate retail pharmacy clinics into a broader national health strategy, focusing on quality assurance, rational prescribing, and longitudinal care. The COVID-19 vaccine rollout in the U.S. successfully leveraged retail clinics and pharmacies, and serves as a precedent for similar partnerships.

We contend that policy innovation in this sector should focus on (1) implementing systems of governance to standardize care delivery through clinical guidelines, (2) creating mechanisms of direct payment to retail pharmacy clinics that incentivize quality over volume and reduce out-of-pocket expenditure, (3) protecting physician autonomy to ensure ethical, patient-centered practice, and (4) ensuring transparency in prescribing practices and pharmaceutical sales.

Retail pharmacy clinics in Mexico address healthcare access gaps but pose systemic risks if allowed to evolve without oversight. As population needs shift and the healthcare market evolves, new opportunities arise to engineer policies that address emerging challenges. The long-term impact of retail pharmacy clinics will be determined not by their existence, but by how they are regulated, integrated, and aligned with national health priorities. Through thoughtful regulation and orchestration of strategic partnerships, we can shift retail pharmacy clinics from a threat to public health to becoming a vital pillar of Mexico’s public healthcare delivery system.

## Contributors

DR: Idea conceptualization and design of the manuscript, realized extensive literature review, co-wrote and edited the manuscript, and was responsible for the decision to submit the manuscript.

MA & GTA: Realized extensive literature review, drafted the manuscript, and provided scientific quality to the manuscript.

## Declaration of interests

The authors declare no competing interests.
